# Taxonomic review of the genus *Teliphasa* Moore, 1888 from China, with descriptions of four new species (Lepidoptera, Pyralidae, Epipaschiinae)

**DOI:** 10.3897/zookeys.554.6177

**Published:** 2016-01-18

**Authors:** Linjie Liu, Yiping Wang, Houhun Li

**Affiliations:** 1 College of Life Sciences, Nankai University, Tianjin 300071, P. R. China

**Keywords:** Lepidoptera, Pyralidae, Epipaschiinae, Teliphasa, taxonomy, new species, China

## Abstract

The genus *Teliphasa* Moore, 1888 from China is reviewed. Nine species are treated, including descriptions of four new species: *Teliphasa spinosa* Li, **sp. n.**, *Teliphasa similalbifusa* Li, **sp. n.**, *Teliphasa erythrina* Li, **sp. n.**, and *Teliphasa hamata* Li, **sp. n.** Photographs of adults, wing venation, and both male and female genitalia are provided, along with a key for the identification of all the Chinese species.

## Introduction

The genus *Teliphasa* was erected by Moore (1888) to accommodate the two species, *Teliphasa orbiculifer* and *Teliphasa nubilosa* from India, with *Teliphasa orbiculifer* as the type. *Teliphasa* is closely allied to *Macalla* Walker, 1859, in which several *Teliphasa* species were once included. Janse (1931) recombined *Stericta picta* Warren, 1895 from *Macalla* to *Teliphasa*, which was later placed in the genus *Orthaga* by Shaffer et al. (1996). Mutuura (1957) transferred *Teliphasa elegans* (Butler, 1881) and *Teliphasa amica* (Butler, 1879) from *Macalla* to *Teliphasa*. Ghesquière (1942) reported *Teliphasa dibelana* from Congo and Zaire, and Viette (1960) described *Teliphasa andrianalis* from Madagascar. Inoue and Yamanaka (1975) made a revision of the *Teliphasa* species in Japan, in which they transfered *Teliphasa albifusa* (Hampson, 1896) from *Macalla* to the present genus and described a new species *Teliphasa sakishimensis*. Solis (1992) provided a list of the Old World Epipaschiinae, in which six species of *Teliphasa* were included. Koçak (1987) proposed a new genus *Sultania* with *Macalla lophotalis* Hampson, 1900 as the type. But currently *Sultania* is considered as a synonym of *Teliphasa* (Nuss et al. 2003–2015).

*Teliphasa* comprises nine species worldwide, occurring in the Oriental, Palaearctic and Ethiopian regions. Five species were recorded in China prior to this study. The aim of the present paper is to review nine species of *Teliphasa* recognized in China, including descriptions of four new species.

## Materials and methods

The present study is based on the examinations of the specimens collected by light traps. Adults were examined using an Olympus SZX9 stereomicroscope. Permanent mounting methods of genitalia and venation follow the techniques introduced by Li (2002). Images of adults and genitalia were taken by using a Leica M205A stereo microscope and a Leica DM750 microscope.

All the studied specimens, including the types of the new species, are deposited in the Insect Collection of College of Life Sciences, Nankai University (NKUM), Tianjin, China.

## Taxonomy

### Genus Teliphasa

Taxon classificationAnimaliaLepidopteraPyralidae

Moore, 1888

Teliphasa Moore, 1888: 200. Type species: *Teliphasa orbiculifer* Moore, 1888.
Sultania Koçak, 1987: 119. Type species: *Macalla lophotalis* Hampson, 1900.

#### Generic characters.

**Figures 1–4.**
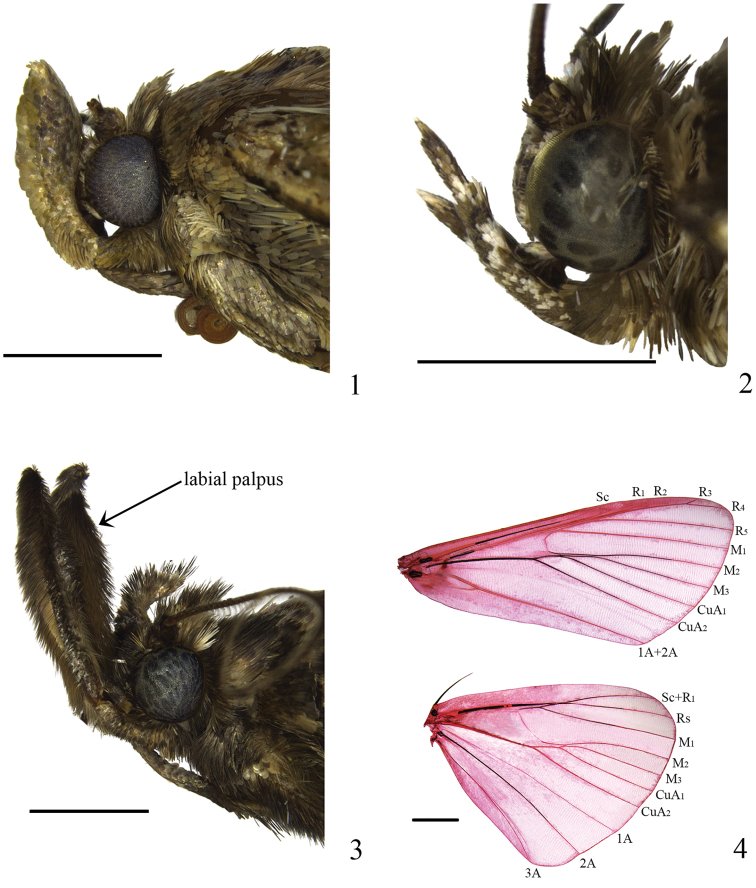
Morphology of *Teliphasa* spp. **1–3** Head **1** *Teliphasa albifusa*, male **2** *Teliphasa nubilosa*, female **3** *Teliphasa nubilosa*, male **4** Venation of *Teliphasa albifusa*, slide No. LLJ15186W. Scale bars: 2.0 mm.

**Adult** (Figs 1–4): Large sized. Head with thick chaetosema. Labial palpus in male (Fig. 1) often stronger than in female (Fig. 2), with diameter of second segment longer than three times length of female, upturned far above vertex of head, even extending back to thorax (Fig. 3), third segment thin, very short, hidden in scales of second; in some species, both male and female labial palpus slender, upturned beyond vertex of head, second segment slightly stronger than third, third segment slender. Antenna thicker in male than in female, male with a row of short cilia along anterior margin. Forewing broad; discal and discocellular spots conspicuous, bearing scale tufts; scale tuft usually set below lower margin of cell near base; antemedian line narrow; postmedian line relatively broad, usually curved outward to form an angle medially; subrectangular spots uniformly placed along inner side of terminal line, interrupted by pale color at veins; hindwing broad triangular, with discocellular spot. Venations (Fig. 4): Forewing with Sc to 2/3 of costa, R_1_ and R_2_ stalked, R_3_ and R_4_ long stalked, R_5_ stalked with R_3+4_, M_1_ from upper angle of cell, M_2_ and M_3_ from lower angle of cell and adjacent in basal 1/4, CuA_1_, CuA_2_ and M_3_ parallel, CuP degenerated, 1A+2A furcated basally; hindwing with Sc+R_1_ and Rs connected at middle of Sc+R_1_, M_2_, M_3_ and CuA_1_ from lower angle of cell, CuA_2_ nearly parallel to CuA_1_.

**Male genitalia.** Uncus various in shape. Gnathos separated, being paired long processes, lateral arms slender. Scaphium columnar, usually narrowed gradually from base to apex. Valva extremely broad, roundly expanded, with numerous long setae. Costa well-developed, varied in shape. Transtilla banded, extending backward to base of uncus, joined medially. Sacculus narrowly banded, sometimes ill-defined. Saccus separated, broad, inverted triangular, or complete, only protruding in short triangle, or ill-defined. Phallus stout, with one or two complicated cornuti.

**Female genitalia.** Apophyses anteriores about same length as apophyses posteriores, occasionally longer than apophyses posteriores. Antrum usually strongly sclerotized. Ductus bursae shorter than or as long as corpus bursae. Corpus bursae pyriform or elliptical; signum paired, often ridged medially.

#### Diagnosis.

This genus is much similar to *Termioptycha* Meyrick, 1889 superficially by having both discal and discocellular spots with scale tufts, the postmedian line relatively broad and curved outward to form an angle medially, and the subrectangular spots set uniformly along the inner side of the terminal line. *Teliphasa* can be easily separated from *Termioptycha* by the costa of forewing without a stigma in the median area, and the hindwing with a discocellular spot; in *Termioptycha*, the costa of forewing has a distinct stigma in the median area, and the hindwing lacks the discocellular spot.

#### Remarks.

Differences within a species exist in *Teliphasa*, including the variations of the wing color and the degree of scale density. For example, *Teliphasa elegans* is divided into a blackish form and a whitish form due to such variations (Inoue and Yamanaka 1975).

#### Key to Chinese species of *Teliphasa* based on male genitalia

**Table:** 

1	Saccus separate; juxta not bilobed	**2**
–	Saccus complete; juxta bilobed	**7**
2	Phallus with two cornuti, one protruding out from before apex of phallus	**3**
–	Phallus with one cornutus	**5**
3	Gnathos hooked apically (Fig. 14)	***Teliphasa similalbifusa* sp. n.**
–	Gnathos rounded apically	**4**
4	Cornutus protruding out from phallus hooked, longer than 1/2 length of phallus (Fig. 16)	***Teliphasa hamata* sp. n.**
–	Cornutus protruding out from phallus horned, slightly longer than 1/3 length of phallus	***Teliphasa sakishimensis***
5	Juxta with clustered spines (Fig. 13)	***Teliphasa spinosa* sp. n.**
–	Juxta without spines	**6**
6	Uncus trapezoidal; costa with a subtriangular process ventrally (Fig. 18)	***Teliphasa albifusa***
–	Uncus subovate; costa without process ventrally (Fig. 19)	***Teliphasa elegans***
7	Uncus triangular (Fig. 15)	***Teliphasa erythrina* sp. n.**
–	Uncus semicircular or irregular in shape	**8**
8	Uncus semicircular; juxta laterally protruding outward semicircularly at base (Fig. 20)	***Teliphasa amica***
–	Uncus irregular in shape; juxta dilated globosely at base (Fig. 17)	***Teliphasa nubilosa***

### Teliphasa spinosa

Taxon classificationAnimaliaLepidopteraPyralidae

Li sp. n.

http://zoobank.org/FCB51905-4D1D-4C16-9841-C1CE835CA26F

Figs 5 , 13 , 21

#### Type material.

Holotype ♂ – **CHINA**, Tengchong County (25.29°N, 98.70°E), Yunnan Province, 2144 m, 15.viii.2014, leg. Kaijian Teng, Shurong Liu and Hua Rong, genitalia slide No. LLJ15172.

Paratypes – 2♂, 2♀, Nankang, Baoshan, Yunnan Province, 2009 m, 8–10.vii.2014, other same date as holotype.

#### Diagnosis.

This species is different from its congeners by the valva triangularly protruding dorso- and ventro-apically and the juxta with clustered spines in the male genitalia. This species is similar to *Teliphasa hamata* sp. n. superficially, but can be differentiated by the subrhombic uncus, the approximately fan-shaped valva, and the phallus with one cornutus in the male genitalia. In *Teliphasa hamata* sp. n., the uncus is trapeziform, the valva is subrhombic, and the phallus has two cornuti in the male genitalia.

#### Description.

**Figures 5–12.**
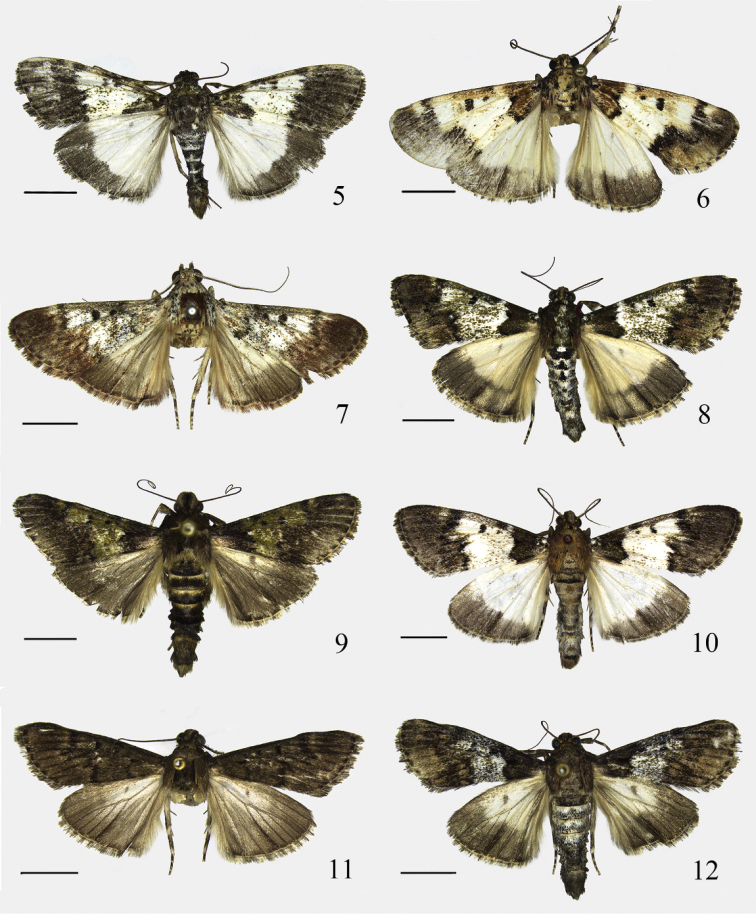
Adults of *Teliphasa* spp. **5** *Teliphasa spinosa* sp. n., paratype, male **6** *Teliphasa similalbifusa* sp. n., holotype, male **7** *Teliphasa erythrina* sp. n., holotype, male **8** *Teliphasa hamata* sp. n., paratype, male **9** *Teliphasa nubilosa*, male **10** *Teliphasa albifusa*, male **11** *Teliphasa elegans*, male **12** *Teliphasa amica*, male. Scale bars: 5.0 mm.

Adult (Fig. 5): Wingspan 34.0–38.0 mm. Head brown, tinged with white scales. Labial palpus blackish brown mixed with white, white at apices of second and third segments; third segment slender, about 1/3 length of second. Maxillary palpus blackish brown, with a few white scales, short, slightly upturned. Antenna with scape brown mottled black, or blackish brown mixed with white; flagellum alternately yellowish brown and deep brown. Thorax and tegula blackish brown, with white scales. Forewing tinged with pale olive-green luster; basal area blackish brown, mixed with black and white scales, with two subrounded white spots near base; median area white, with scattered pale brown and blackish brown scales, with dense brown and blackish brown scales from costa diffused to above cell, forming a narrow elongate dark streak; distal area deep brown, with black scales; costa with a white spot at outside of postmedian line, spreading to R_5_, mixed with pale brown; antemedian line black, extending from costal 1/4 obliquely inward to scale tuft near base, then obliquely outward to 1/3 on dorsum; postmedian line black, extending from costal 2/3 slightly oblique outward to R_5_, then running slightly oblique inward to dorsal 2/3, its inner margin more or less serrated; discal spot almost circular, smaller than discocellular spot; discocellular spot nearly trapeziform; terminal line white, with ill-defined subrectangular black spots uniformly placed along its inner side, interrupted by grayish white mixed with blackish brown or brown scales at veins; cilia yellowish brown to brown, blackish brown along extension of veins. Hindwing with basal 3/4 white, distal 1/4 deep brown; discocellular spot pale grayish brown; cilia yellowish brown or brown. Legs brownish yellow, mixed with white, brown and black scales; tarsi with each tarsomere white apically, except black at apex of last tarsomere. Abdomen blackish brown with white, intersegment white.

**Figures 13–20.**
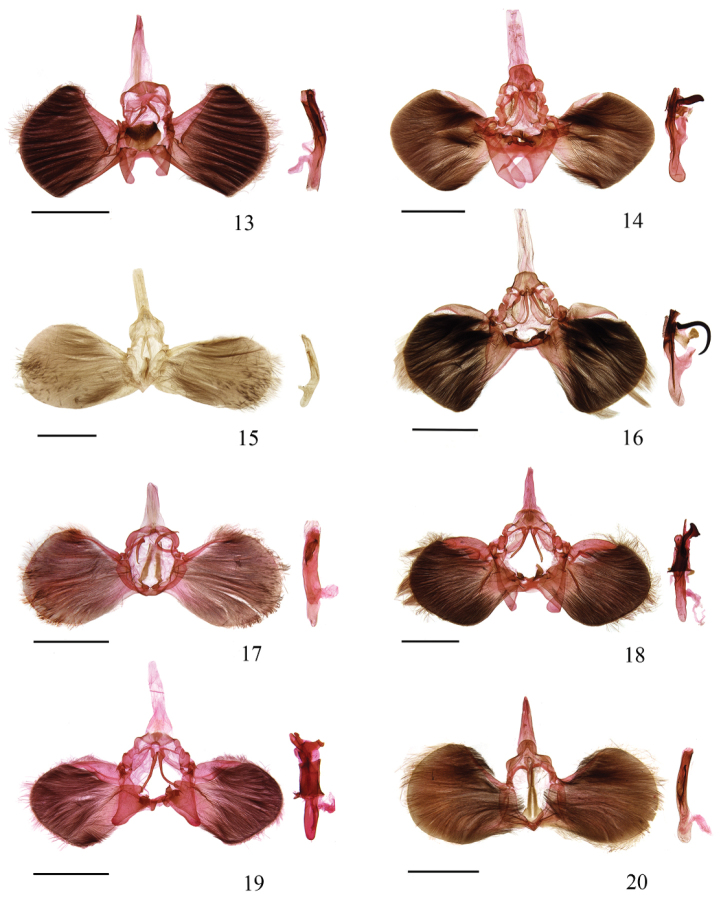
Male genitalia of *Teliphasa* spp. **13** *Teliphasa spinosa* sp. n., holotype, slide No. LLJ15172 **14** *Teliphasa similalbifusa* sp. n., holotype, slide No. LLJ13039 **15** *Teliphasa erythrina* sp. n., holotype, slide No. WYP05198 **16** *Teliphasa hamata* sp. n., holotype, slide No. LLJ15167 **17** *Teliphasa nubilosa*, slide No. LLJ15173 **18** *Teliphasa albifusa*, slide No. LLJ15175 **19** *Teliphasa elegans*, slide No. LLJ13044 **20** *Teliphasa amica*, slide No. LLJ15178. Scale bars: 2.0 mm.

**Male genitalia** (Fig. 13). Uncus transversally subrhombic. Gnathos slightly dilated in basal 1/3, then gradually narrowed to gently hooked apex; about 1/3 length of scaphium. Valva approximately fan-shaped, triangularly protruding dorso- and ventro-apically. Costa narrow, elongate triangular, reaching valva apically. Sacculus narrowly banded, wide basally, narrowed distally, reaching ventral 4/5 length of valva. Transtilla joined medially in a knot. Juxta nearly circular, heavily sclerotized, with clustered spines. Saccus separated. Phallus curved at middle; cornutus a long plate, narrow basally, serrated along dorsal margin of distal half and on apex.

**Figures 21–26.**
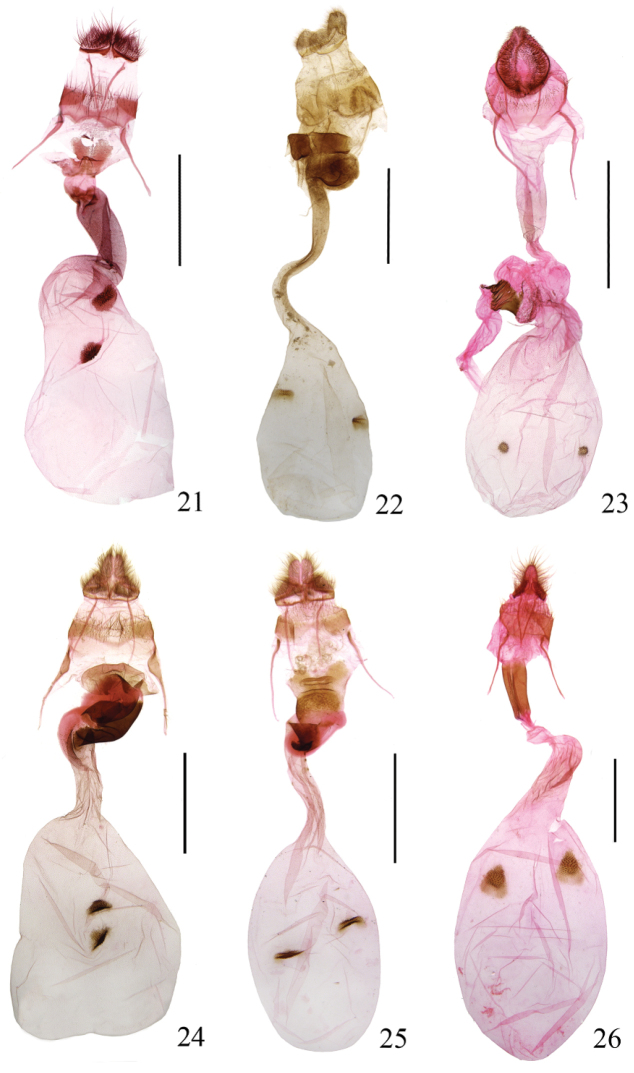
Female genitalia of *Teliphasa* spp. **21** *Teliphasa spinosa* sp. n., paratype, slide No. RH15180 **22** *Teliphasa hamata* sp. n., paratype, slide No. WYP06011 **23** *Teliphasa nubilosa*, slide No. LLJ15182 **24** *Teliphasa albifusa*, slide No. LLJ15174 **25** *Teliphasa elegans*, slide No. LLJ13038 **26** *Teliphasa amica*, slide No. LLJ13017. Scale bars: 2.0 mm.

**Female genitalia** (Fig. 21). Papillae anales subrectangular, densely setose. Eighth tergite weakly sclerotized at middle posteriorly, with setae, anterior margin wavy, obviously concave medially; eighth sternite produced to paired triangular plates, separated medially, strongly sclerotized. Apophyses anteriores about same length as apophyses posteriores, dilated basally, gradually thinned distally, thicker than apophyses posteriores. Antrum subquadrate, slightly thicker than ductus bursae. Ductus bursae stout, strongly sclerotized, with clustered granules on inner surface; about 2/5 length of corpus bursae. Corpus bursae pyriform; signum semicircular, strongly sclerotized, with dense spines.

#### Distribution.

China (Yunnan).

#### Etymology.

The specific name is derived from the Latin *spinosus* (with many spines), in reference to the juxta with clustered spines in the male genitalia.

#### Remarks.

The labial palpus of this species is not sexual dimorphic. The shape and pattern of wing is consistent with the diagnostic characters of *Teliphasa*, and the male genitalia resemble other congeners in having a paired gnathos, the roundly expanded valva, and the separated inverted triangular saccus. Therefore, we regard this species as a new species of *Teliphasa* and describe it here.

### Teliphasa similalbifusa

Taxon classificationAnimaliaLepidopteraPyralidae

Li sp. n.

http://zoobank.org/2BC387B1-E606-4E4C-989F-7487507242CF

Figs 6 , 14

#### Type material.

Holotype ♂ – **CHINA**, Mt. Daming (23.40°N, 108.48°E), Guangxi Zhuang Autonomous Region, 1250 m, 20.v.2011, leg. Linlin Yang and Yinghui Mou, genitalia slide No. LLJ13039.

#### Diagnosis.

This species is similar to *Teliphasa albifusa* superficially, but can be separated from the latter by the gnathos about 3/5 the length of the scaphium, the costa without process near base on the ventral margin, and the phallus with two cornuti in the male genitalia. In *Teliphasa albifusa*, the gnathos is about 3/4 the length of the scaphium, the costa is protruding subtriangularly near base on the ventral margin, and the phallus has a single cornutus in the male genitalia. In addition, *Teliphasa similalbifusa* sp. n., *Teliphasa hamata* sp. n. and *Teliphasa sakishimensis* are much similar in the male genitalia by the subtrapeziform uncus, the approximately rhombic valva, the broadly banded transtilla, the irregularly shaped juxta, the separated saccus, and one of the paired cornuti stretching out from before the apex of the phallus. However, the gnathos is hooked apically, and the stretching out cornutus is stout and shorter than 1/3 the length of the phallus in *Teliphasa similalbifusa* sp. n.; the gnathos is obtuse apically, and the stretching out cornutus is slender and longer than 1/2 the length of the phallus in *Teliphasa hamata* sp. n.; the gnathos is also obtuse apically, but the stretching out cornutus is horned and slightly longer than 1/3 the length of the phallus in *Teliphasa sakishimensis*.

#### Description.

Adult (Fig. 6): Wingspan 34.0 mm. Head white, mixed with yellowish brown and black scales. Male labial palpus white mixed with dense yellowish brown; second segment strong; third segment short, apex pointed. Antenna with scape white, mixed with yellowish brown and black scales; flagellum alternately pale yellowish brown and deep brown. Thorax white, with scattered black and brown scales. Tegula blackish brown, mixed with white scales, with long brown scales distally. Forewing with basal area pale ocherous brown, mixed with black and white scales; median area white mixed with pale yellow and yellowish brown scales, with dense yellowish brown scales from costa scattered to above cell; distal area yellowish brown, mixed with black scales; antemedian line black, ill-defined anteriorly, extending distinctly from black scale tuft near base obliquely outward to below 1A+2A, then straightly reaching dorsal 1/3; postmedian line black, extending from costal 3/4 obliquely outward to M_2_, then arched and extending inward along CuA_1_, forming a blunt angle, finally straight to dorsal 2/3, its inner margin serrated; costa with a blackish brown spot at basal 1/3 diffused to above cell, with a white spot at outside of postmedian line spreading to R_5_; discal spot small, black surrounded by pale yellowish brown, with raised white scales on its outer margin; discocellular spot nearly rectangular, relatively large, surrounded by pale yellowish brown scales; terminal line yellowish white, spots ill-defined along its inner side; cilia yellowish white mixed with pale brown, blackish brown along extension of veins. Hindwing with basal 3/4 white mixed with pale yellow; distal 1/4 grayish brown, deepening from costa to dorsum; discocellular spot pale grayish brown; cilia yellowish white, blackish brown along extension of veins, with a brown line near base. Legs yellowish white, mixed with blackish brown and grayish white scales; tarsi blackish brown, white at apex of each tarsomere except black at apex of last tarsomere.

**Male genitalia** (Fig. 14). Uncus nearly trapeziform, rounded on posterior margin. Gnathos slightly dilated at base, gradually narrowed to hooked apex; about 3/5 length of scaphium. Valva approximately rhombic. Costa narrow, elongate triangular, smoothly arched dorsally, reaching before apex of valva. Sacculus narrowly banded, not reaching apex of valva. Transtilla joined in a semicircular protrusion. Juxta irregular in shape, heavily sclerotized. Saccus separated. Phallus dilated in basal 1/5, protruding at dorsal 1/2, relatively narrow distally; two cornuti present: one a long plate, serrated along dorsal margin distally; another stout, heavily sclerotized, stretching out of phallus from before apex, shorter than 1/3 length of phallus, narrow basally, broad distally, hooked apically.

Female unknown.

#### Distribution.

China (Guangxi).

#### Etymology.

The specific name is derived from the Latin *simil*- (similar), and the specific name of another species *albifusa*, in reference to the similarity of the two species in the superficial morphology.

### Teliphasa erythrina

Taxon classificationAnimaliaLepidopteraPyralidae

Li sp. n.

http://zoobank.org/B721CC89-7816-44E0-9FFC-C0CFC9CF208B

Figs 7 , 15

#### Type material.

Holotype ♂ – **CHINA**, Bubang, Mengla County (21.60°N, 101.59°E), Yunnan Province, 650 m, 23.viii.2005, leg. Yingdang Ren, genitalia slide No. WYP05198.

#### Diagnosis.

This species is obviously different from its congeners by the forewing reddish brown and the hindwing deep gray tinged with pale reddish brown in the distal area. In addition, this species is distinguishable by the subtriangular uncus and the relatively narrow valva with length obviously longer than the maximum width in the male genitalia. In other *Teliphasa* species, the uncus is not subtriangular, and the relatively broad valva is shorter than or equal to the maximum width.

#### Description.

Adult (Fig. 7): Wingspan 36.0 mm. Head white, mixed with pale yellow and blackish brown scales. Male labial palpus grayish white, with scattered brown scales; second segment dilated near base; third segment shorter than 1/10 length of second. Antenna with scape white, mixed with brown; flagellum alternately yellowish brown and brown. Thorax and tegula white, mixed with brownish yellow and black scales; tegula with brown long scales distally. Forewing white in basal 2/3, suffused with pale yellowish, pale brown and brownish black scales, with blackish brown scale tuft near base, mottled white; costa with brownish black scales from basal 1/3 to 2/3, diffused to above cell, with a white spot at outside of postmedian line, spreading to R_5_; distal 1/3 reddish brown, tinged with white scales; antemedian line black wavy, extending from costal 1/3 obliquely outward to below cell, then obliquely inward to scale tuft near base, thereafter obliquely outward to 1A+2A, finally straight to dorsal 1/3; postmedian line black, extending from costal 2/3 obliquely outward to M_3_, then running obliquely inward to dorsal 2/3, its inner margin serrated; discal and discocellular spots brownish black, the former circular, the latter subrectangular; terminal line grayish white, with subrectangular blackish brown spots uniformly placed along its inner side, interrupted by pale brown at veins. Hindwing with basal 2/3 white mixed with grayish scales; distal 1/3 deep gray tinged with pale reddish brown, gradually paler from costa to dorsum; discocellular spot pale brown; cilia of fore- and hind-wings brownish yellow, blackish brown along extension of veins. Legs yellowish white, mixed with blackish brown and pale brown scales; tarsi blackish brown, white at apex of each tarsomere.

**Male genitalia** (Fig. 15). Uncus triangular, concave medially on anterior margin. Gnathos gradually narrowed to gently hooked apex; about 3/5 length of scaphium. Valva relatively narrow, length about three times width at base. Costa narrowly banded, reaching valva apically. Transtilla joined medially in a small knot. Sacculus narrowly banded, reaching 1/2 length of valva on ventral margin. Juxta bilobed, each lobe gradually narrowed distally, inner side strongly sclerotized. Saccus not separated, protruding triangularly, acute apically. Phallus slender, slightly curved at basal 1/3; cornutus broad basally, slightly curved medially, dilated distally, protruding to a stout hook laterally.

Female unknown.

#### Distribution.

China (Yunnan).

#### Etymology.

The specific name is derived from the Latin *erythrinus* (red), in reference to the forewing reddish brown in the distal area.

### Teliphasa hamata

Taxon classificationAnimaliaLepidopteraPyralidae

Li sp. n.

http://zoobank.org/CCF9D60B-7574-46FC-8012-1EA281C2EA54

Figs 8 , 16 , 22

#### Type material.

Holotype ♂ – **CHINA**, Tengchong County (25.29°N, 98.70°E), Yunnan Province, 2144 m, 16.viii.2014, leg. Kaijian Teng, Shurong Liu and Hua Rong, genitalia slide No. LLJ15172.

Paratypes: 2♂, same data as holotype; 1♂, 2♀, Wenshan County, 1105 m, xi.2003, leg. Shengxian Lu; 2♂, Kunming, Yunnan Province, 1900 m, 29.viii.2005, leg. Yingdang Ren; 1♂, Mt. Jizu, Dali, Yunnan Province, 2228 m, 27.vii.2014, leg. Kaijian Teng et al.; 1♂, Xianfengling, Mt. Wuyi, Fujian Province, 1000 m, 26.v.2004, leg. Haili Yu; 1♂, 1♀, Huaping, Leyie County, Guangxi Zhuang Autonomous Region, 1300 m, 6, 8.viii.2006, leg. Weichun Li; 3♂, 3♀, Mt. Yuanbao, Guangxi Zhuang Autonomous Region, 700 m, 11.viii.2006, leg. Weichun Li.

#### Diagnosis.

This species is similar to *Teliphasa albifusa* superficially in the forewing color, but can be distinguished by the gnathos about 2/3 the length of the scaphium and the phallus with two cornuti in the male genitalia. In *Teliphasa albifusa*, the gnathos is about 3/4 the length of the scaphium, and the phallus has a single cornutus in the male genitalia. Moreover, *Teliphasa hamata* sp. n., *Teliphasa similalbifusa* sp. n., and *Teliphasa sakishimensis* are extremely similar in the male genitalia, and differences between them are stated under *Teliphasa similalbifusa* sp. n.

#### Description.

Adult (Fig. 8): Wingspan 32.0–36.0 mm. Head white, suffused with black and pale brown scales. Male labial palpus pale yellow to brownish yellow, suffused with white and black scales; second segment slightly dilated in distal 2/3; third segment short, pointed at apex; female labial palpus with second segment white, mixed with yellowish brown scales on dorsal surface; third segment slender, yellowish brown with black, about 1/5 length of second. Antenna with scape white mottled blackish brown; flagellum alternately grayish brown and blackish brown. Thorax white, with brown scales. Tegula deep brown, with black and a few white scales. Forewing with basal area dark brown, mixed with black scales; median area white, with scattered brown scales; distal area ochreous brown, mixed with black and white scales; costa brown from basal 1/3 to 2/3, mixed with black scales, diffused to above cell, with a white spot near base and raised black scales at its outside, with a second white spot at outside of postmedian line, spreading to below R_5_; antemedian line black, extending from costal 1/4 obliquely inward to scale tuft near base, then obliquely outward to below 1A+2A, finally obliquely inward to dorsal 1/3; postmedian line black, extending from costal 2/3 obliquely outward to M_2_, then curved and extending inward along CuA_1_, forming an angle, finally straight to dorsal 2/3, its inner margin serrated; discal and discocellular spots black, the latter relatively large; terminal line pale yellow, with subrectangular brown or blackish brown spots uniformly placed along its inner side, interrupted by white mixed with brown at veins. Hindwing with basal 2/3 yellowish white, distal 1/3 grayish brown, becoming paler from costa to dorsum; discocellular spot pale grayish brown; cilia of fore- and hind-wings pale yellow, grayish brown to brownish yellow along extension of veins. Legs yellowish brown to blackish brown, mixed with black, brown and a few white scales; tarsi blackish brown, white at apex of each tarsomere. Abdomen white mottled black scales except first segment black.

**Male genitalia** (Fig. 16). Uncus broad, nearly trapeziform, rounded on posterior margin. Gnathos slender, basal half thicker than distal half, apex obtuse; about 2/3 length of scaphium. Valva subrhombic, with relatively dense setae near costa. Costa elongate triangular, broad basally, gradually narrowed distally, reaching before apex of valva; distal 1/4 obviously thinned, slightly curved inward in a hook. Sacculus narrowly banded, reaching valva apically. Transtilla knotted medially as a semielliptical expansion. Juxta irregular in shape, heavily sclerotized. Saccus separated. Phallus protruding near dorsal 1/2; with two cornuti, one a long plate, serrated along dorsal margin of distal part, another slender, heavily sclerotized, protruding out from phallus near apex, longer than 1/2 length of phallus, curved medially and forming a large hook.

**Female genitalia** (Fig. 22). Papillae anales subtriangular, densely setose. Eighth tergite nearly rectangular, with sparse setae posteriorly; eighth sternite paired triangular plates. Apophyses anteriores about same length as apophyses posteriores; thicker than apophyses posteriores. Antrum rectangular, heavily sclerotized. Ductus bursae thin, slightly longer than corpus bursae, weakly sclerotized posteriorly, with one horned plate basally. Corpus bursae pyriform; signum nearly rectangular, with clustered spines.

#### Distribution.

China (Fujian, Guangxi, Yunnan).

#### Etymology.

The specific name is derived from the Latin *hamatus* (hooked), referring to the slender hooked cornutus protruding out from the phallus in the male genitalia.

### Teliphasa nubilosa

Taxon classificationAnimaliaLepidopteraPyralidae

Moore, 1888

Figs 9 , 17 , 23

Teliphasa nubilosa Moore, 1888: 201. Type Locality: India (Darjiling).
Macalla formisibia Strand, 1919: 50.

#### Material examined.

**CHINA: Fujian Province**: 9♂, Xianfengling, Mt. Wuyi, 1000 m, 26.v.2004, leg. Haili Yu; **Guangxi Zhuang Autonomous Region**: 10♂, 2♀, Huaping, Leyie County, 1300 m, 1, 8.viii.2006, leg. Weichun Li; 2♂, Mt. Yuanbao, 700 m, 12.viii.2006, leg. Weichun Li; 5♂, 6♀, Mt. Mao’er, Guilin, 1016 m, 23–24.vii.2015, leg. Mujie Qi and Shengnan Zhao; 4♂, Huanjiang County, Hechi, 1180 m, 23–26.vii.2015, leg. Meiqing Yang and Ga-Eun Lee; **Guizhou Province**: 1♂, Limingguan, Libo County, 720 m, 19.vii.2015, leg. Meiqing Yang and Ga-Eun Lee; **Hainan Province**: 3♂, Mt. Wuzhi, 700 m, 19.v.2007, leg. Zhiwei Zhang and Weichun Li; 2♂, Mt. Diaoluo, 940 m, 31.v.2007, leg. Zhiwei Zhang and Weichun Li; 2♂, Yinggeling, Qiongzhong County, 508 m, 27.vii.2014, leg. Peixin Cong, Linjie Liu and Sha Hu; 2♀, Yuanmenxiang, Baisha County, 460 m, 29–30.vi.2014, leg. Peixin Cong, Linjie Liu and Sha Hu; 1♂, Jianfengling, Ledong County, 770 m, 14.vii.2014, leg. Peixin Cong, Linjie Liu and Sha Hu; 5♂, Mt. Limu, 607 m, 25, 27.vii.2014, leg. Peixin Cong, Linjie Liu and Sha Hu; 5♂, Mt. Wuzhi, 742 m, 20.V.2015, leg. Peixin Cong, Wei Guan and Sha Hu; **Henan Province**: 2♂, 3♀, Lushi County, 1560 m, 19, 21.vii.2001, leg. Dandan Zhang; 1♂, 2♀, Mt. Baiyun, Luoyang County, 1560 m, 22–24.vii.2001, leg. Dandan Zhang; 157♂, 40♀, Mt. Yuntai, Jiaozuo, 1028 m, 5–12.viii.2014, leg. Peixin Cong, Linjie Liu and Sha Hu; **Hubei Province**: 1♀, Pingbaying, Xianfeng, 1280 m, 22.vii.1999, leg. Houhun Li; **Jiangxi Province**: 3♂, Mt. Jiulian, 20–21.vii.2006, leg. Weichun Li; **Sichuan Province: 1**♂, Qingyinge, Mt. Emei, 900 m, 30.iv.1957, leg. Leyi Zheng and Hanhua Cheng; 3♀, Mabian County, 900 m, 21.vii.2004, leg. Yingdang Ren; 2♂, Wenchuan County, 1557 m, 9.vii.2014, leg. Kaijian Teng et al.; 1♂, Wanniansi, Mt. Emei, 830 m, 14.vii.2014, leg. Kaijian Teng et al.; **Yunnan Province**: 1♂, Bubang, Mengla County, 650 m, 23.viii.2005, leg. Yingdang Ren; 1♂, Taiyanghe, Pu’er, 1626 m, 8.vii.2013, leg. Shurong Liu, Yuqi Wang and Kaijian Teng; 1♂, Mt. Jizu, Dali, 2228 m, 27.vii.2014, leg. Kaijian Teng et al.; 10♂, Mt. Weibao, Dali, 2205 m, 30.vii.2014, leg. Kaijian Teng et al.; 1♂, Xiaodifang, Tengcong County, 2116 m, 11.viii.2014, leg. Kaijian Teng, Shurong Liu and Hua Rong; 1♂, Linjiapu, Tengcong County, 2144 m, 16.viii.2014, leg. Kaijian Teng, Shurong Liu and Hua Rong; **Zhejiang Province**: 5♂, Xianrending, Mt. Tianmu, 1500 m, 25.vii.2011, leg. Xicui Du and Xiaobing Fu; 1♂, Laoan, Mt. Tianmu, 555 m, 3.vii.2014, leg. Aihui Yin, Xuemei Hu and Qingyun Wang; 16♂, Sanmuping, Mt. Tianmu, 789 m, 13–15.vii.2014, leg. Aihui Yin, Xuemei Hu and Qingyun Wang; 2♂, Xiguan, Mt. Tianmu, 566 m, 16, 18.vii.2014, leg. Aihui Yin, Xuemei Hu and Qingyun Wang; 9♂, Mt. Longxu, 754 m, 20–22.vii.2014, leg. Aihui Yin, Xuemei Hu and Qingyun Wang; 2♂, Qingliangfeng, 1059 m, 28.vii.2014, leg. Aihui Yin, Xuemei Hu and Qingyun Wang; 5♂, 20♀, Sanmuping, Mt. Tianmu, 789 m, 16–17.vii.2015, leg. Aihui Yin, Kang Lou and Tao Wang.

#### Diagnosis.

This species is different from its congeners by the forewing suffused with olive-green scales in the median area, the rather thick male labial palpus extending to thorax, and the dorsal side of the labial palpus with long brownish yellow hairs in the distal 3/4. In addition, this species is similar to *Teliphasa amica* in the male genitalia (Fig. 17) by the valva nearly circular, the costa narrowly banded, and the juxta with lobes. It can be distinguished by the narrow uncus irregular in shape, the juxta dilated globosely at base, the saccus ill-defined, and the cornutus about 1/4 the length of the phallus in the male genitalia; the signum set at anterior 1/3 of the corpus bursae in the female genitalia (Fig. 23). In *Teliphasa amica*, the uncus is semicircular, the juxta is protruding semicircularly at base laterally, the distinct saccus produced in a short triangle, the cornutus is about 1/2 the length of the phallus in the male genitalia; the signum is located at posterior 1/4 of the corpus bursae in the female genitalia.

#### Description.

Adult (Fig. 9): Wingspan 26.0–38.0 mm.

#### Distribution.

China (Fujian, Guangxi, Guizhou, Hainan, Henan, Hubei, Jiangxi, Sichuan, Yunnan, Zhejiang, Taiwan), India.

### Teliphasa albifusa

Taxon classificationAnimaliaLepidopteraPyralidae

(Hampson, 1896)

Figs 10 , 18 , 24

Macalla albifusa Hampson, 1896: 113. Type Locality: Sikkim, Nagas.
Teliphasa albifusa (Hampson): Inoue and Yamanaka 1975: 99.

#### Material examined.

**CHINA: Guangxi Zhuang Autonomous Region**: 1♂, 2♀, Mt. Mao’er, Guilin, 1016 m, 23.vii.2015, leg. Mujie Qi and Shengnan Zhao; 2♂, 1♀, Huanjiang County, Hechi, 1180 m, 25.vii.2015, leg. Meiqing Yang and Ga-Eun Lee; 2♂, Rongshui County, Liuzhou, 1240 m, 27.vii.2015, leg. Meiqing Yang and Ga-Eun Lee; **Hebei Province**: 10♂, 3♀, Xinglong County, 800 m, 16–29.vii.2011, leg. Houhun Li and Yanpeng Cai; **Henan Province**: 1♀, Mt. Baiyun, Luoyang, 1560 m, 22.vii.2001, leg. Dandan Zhang; 14♂, 8♀, Linzhou, 550 m, 21, 23.vii.2006, leg. Hui Zhen and Denghui Kuang; 1♂, Mt. Guan, Hui County, 550 m, 26.vii.2006, leg. Hui Zhen and Denghui Kuang; 3♂, 1♀, Mt. Wangwu, Jiyuan, 800 m, 28–29.vii.2006, leg. Hui Zhen and Denghui Kuang; 15♂, 4♀, Mt. Yuntai, Jiaozuo, 1028 m, 5–10.viii.2014, leg. Peixin Cong, Linjie Liu and Sha Hu; **Hubei Province**: 3♂, 3♀, Shennongjia, 15.vii.1977, leg. Leyi Zheng; 5♂, 1♀, Pingbaying, Xianfeng, 1280 m, 21–22.vii.1999, leg. Houhun Li et al.; 1♂, Lichuan, 700 m, 28.vii.1999, leg. Houhun Li et al.; **Sichuan Province**: 2♂, Baoxing County, 900 m, 1.viii.2004, leg. Yingdang Ren; 3♂, 2♀, Wenchuan County, 1557 m, 11.vii.2014, leg. Kaijian Teng et al.; 1♀, Wanniansi, Mt. Emei, 830 m, 14.vii.2014, leg. Kaijian Teng et al.; **Shanxi Province**: 3♂, Manghe, Yangcheng County, 594 m, 13, 16.vii.2012, leg. Wei Guan and Xiuchun Wang; **Tianjin**: 1♂, Mt. Baxian, 550 m, 23.vi.2001, leg. Houhun Li et al.; 9♂, 3♀, Mt. Baxian, 560 m, 14, 16.vii.2005, leg. Houhun Li et al.; 2♂, Mt. Baxian, 550 m, 24.vii.2015, leg. Houhun Li and Peixin Cong; **Yunnan Province**: 1♂, Bubang, Mengla County, 650 m, 22.viii.2005, leg. Yingdang Ren; 1♂, Yexianggu, Xishuangbannan, 762 m, 19.vii.2014, leg. Kaijian Teng et al.; **Zhejiang Province**: 1♂, Wuyanling, Taishun, 680 m, 31.vii.2005, leg. Yunli Xiao; 27♂, 2♀, Laoan, Mt. Tianmu, 555 m, 3–6.vii.2014, leg. Aihui Yin, Xuemei Hu and Qingyun Wang; 2♂, 1♀, Qianjiangyuan, Mt. Tianmu, 866 m, 7, 10.vii.2014, leg. Aihui Yin, Xuemei Hu and Qingyun Wang; 11♂, Xiguan, Mt. Tianmu, 566 m, 16–20.vii.2014, leg. Aihui Yin, Xuemei Hu and Qingyun Wang; 4♂, Mt. Longxu, 754 m, 20–22.vii.2014, leg. Aihui Yin, Xuemei Hu and Qingyun Wang; 3♂, Mt. Longtang, 520 m, 28, 30.vii.2014, leg. Aihui Yin, Xuemei Hu and Qingyun Wang; 9♂, Sanmuping, Mt. Tianmu, 789 m, 30.viii.2014, leg. Aihui Yin, Xuemei Hu and Qingyun Wang; 20♂, 16♀, Sanmuping, Mt. Tianmu, 789 m, 16–17.vii.2015, leg. Aihui Yin, Kang Lou and Tao Wang.

#### Diagnosis.

This species is characterized by the subtrapeziform uncus, the gnathos about 3/4 the length of the scaphium, the transtilla joined medially in a semicircular knot, the juxta irregular in shape, the phallus ventrally with a short slender process distally and with a row of spines internally in the male genitalia (Fig. 18); the ductus bursae with two irregular sclerotized plates posteriorly, and the triangular signum in the female genitalia (Fig. 24). *Teliphasa albifusa* is similar to *Teliphasa similalbifusa* sp. n. and the whitish form of *Teliphasa elegans* superficially, and the differences between them are stated under *Teliphasa similalbifusa* sp. n. and *Teliphasa elegans*.

#### Description.

Adult (Fig. 10): Wingspan 34.0–38.0 mm.

#### Distribution.

China (Fujian, Guangxi, Hebei, Henan, Hubei, Hunan, Shanxi, Sichuan, Tianjin, Yunnan, Zhejiang, Taiwan), Japan, Korea, Sikkim, Nagas.

### Teliphasa elegans

Taxon classificationAnimaliaLepidopteraPyralidae

(Butler, 1881)

Figs 11 , 19 , 25

Locastra elegans Butler, 1881: 581. Type Locality: Japan (Yokohama).
Macalla elegans (Butler): Rebel 1901: 258.
Teliphasa elegans (Butler): Mutuura 1957: 105.

#### Material examined.

**CHINA: Guizhou Province**: 2♂, Suoluo, Chishui, 390 m, 27–28.V.2000, leg. Yanli Du; **Hebei Province**: 4♂, Xinglong County, 800 m, 20.vii.2011, leg. Houhun Li and Yanpeng Cai; **Heilongjiang Province**: 2♀, Mt. Mao’er, 18.vii.2009, leg. Weichun Li and Jiayu Liu; **Henan Province**: 1♀, Mt. Jigong, Xinyang, 700 m, 13.vii.2001, leg. Dandan Zhang; 1♀, Shiziping, Lushi County, 1200 m, 21.vii.2001, leg. Dandan Zhang; 1♂, Baligou, Huixian, 780 m, 12.vii.2002, leg. Xinpu Wang; **Hubei Province**: 1♂, Shennongjia, 15.vii.1977, leg. Leyi Zheng; **Tianjin**: 3♂, Mt. Baxian, 560 m, 13–14.vii.2005, leg. Houhun Li et al.; 1♀, Mt. Jiulong, 10.vii.2009, leg. Weichun Li.

#### Diagnosis.

This species has two forms: the blackish form and the whitish from. The blackish form can be differentiated by the forewing and the distal 2/3 of hindwing blackish brown. The whitish form is similar to *Teliphasa albifusa* superficially, but can be separated from the latter by the uncus subovate, the costa without process ventrally, and the phallus ventrally with two short digitiform distal processes that lack spines internally in the male genitalia (Fig. 19); the ductus bursae with one sclerotized subtriangular plate posteriorly, and the signum narrow rectangular in the female genitalia (Fig. 25). Whereas in *Teliphasa albifusa*, the uncus is trapezoidal, the costa has a subtriangular process ventrally, and the phallus ventrally with a short slender distal process that has a row of spines internally in the male genitalia; the ductus bursae with two irregular sclerotized plates posteriorly, and the signum is subtriangular in the female genitalia.

#### Description.

Adult (Fig. 11): Wingspan 34.0–38.0 mm.

#### Host plants.

*Glycine max* (Linn.) Merr. (Song and He 1977), *Cornus macrophylla* Wall. (Hayashi 2006).

#### Distribution.

China (Fujian, Guangxi, Guizhou, Hebei, Heilongjiang, Henan, Hubei, Hunan, Shaanxi, Tianjin), Korea, Japan, Ussuri.

### Teliphasa amica

Taxon classificationAnimaliaLepidopteraPyralidae

(Butler, 1879)

Figs 12 , 20 , 26

Locastra amica Butler, 1879: 447. Type Locality: Japan.
Macalla amica (Butler): Hampson 1896: 454.
Teliphasa amica (Butler): Mutuura 1957: 105.

#### Material examined.

**CHINA: Guangxi Zhuang Autonomous Region**: 1♀, Huaping, Leyie County, 950 m, 8.viii.2006, leg. Weichun Li; 1♂, 1♀, Mt. Yuanbao, 700 m, 11.viii.2006, leg. Weichun Li; **Hebei Province**: 4♂, 1♀, Xinglong County, 800 m, 20.vii.2011, leg. Houhun Li and Yanpeng Cai; **Henan Province**: 1♂, 1♀, Mt. Jigong, Xinyang, 700 m, 13.vii.2001, leg. Dandan Zhang; 1♀, Mt. Tongbai, Tongbai County, 300 m, 16.vii.2001, leg. Dandan Zhang; 35♂, 9♀, Linzhou, 550 m, 21, 23.vii.2006, leg. Hui Zhen and Denghui Kuang; 4♂, Mt. Guan, Hui County, 550 m, 26.vii.2006, leg. Hui Zhen and Denghui Kuang; 31♂, 14♀, Mt. Wangwu, Jiyuan, 800 m, 28–30.vii.2006, leg. Hui Zhen and Denghui Kuang; 4♂, Mt. Yuntai, Jiaozuo, 1028 m, 10.viii.2014, leg. Peixin Cong, Linjie Liu and Sha Hu; **Hubei Province**: 1♂, 1♀, Shennongjia, 1977, leg. Huanguang Zou; **Jiangxi Province**: 1♀, Mt. Jiulong, 10.vii.2009, leg. Weichun Li; 1♀, Mt. Jiulian, 21.vii.2006, leg. Weichun Li; **Sichuan Province**: 1♂, Qingyinge, Mt. Emei, 900 m, 30.iv.1957, leg. Leyi Zheng and Hanhua Cheng; **Tianjin**: 4♂, Mt. Baxian, 550 m, 23–24.vi.2001, leg. Houhun Li et al.; 1♂, 1♀, Mt. Pan, Ji County, 170 m, 20.vii.2004, leg. Houhun Li et al.; 19♂, 6♀, Mt. Baxian, 560 m, 13, 16.vii.2005, leg. Houhun Li et al.; 3♂, 1♀, Mt. Baxian, 550 m, 23–24.vii.2015, leg. Houhun Li and Peixin Cong; **Zhejiang Province**: 2♂, Qingliangfeng, Linan, 900 m, 10, 12.viii.2005, leg. Yunli Xiao; 1♂, Xiguan, Mt. Tianmu, 566 m, 17.vii.2014, leg. Aihui Yin, Xuemei Hu and Qingyun Wang; 1♂, Yulingguan, Qingliangfeng, 220 m, 24.vii.2014, leg. Aihui Yin, Xuemei Hu and Qingyun Wang; 1♂, Mt. Longtang, 520 m, 26.vii.2014, leg. Aihui Yin, Xuemei Hu and Qingyun Wang; 2♂, Qianqingtang, Qingliangfeng, 1059 m, 28.vii.2014, leg. Aihui Yin, Xuemei Hu and Qingyun Wang; 2♂, Sanmuping, Mt. Tianmu, 789 m, 8.viii.2014, leg. Aihui Yin, Qingyun Wang and Suran Li.

#### Diagnosis.

This species is characterized by having a semicircular uncus, the gnathos about 1/2 the length of the scaphium, the transtilla medially produced to a rectangular extension, the bilobed juxta in the male genitalia (Fig. 20); the elongate rectangular antrum and the triangular signum in the female genitalia (Fig. 26). *Teliphasa amica* is similar to *Teliphasa hamata* sp. n. superficially, but distinguishable in the male genitalia by the saccus completed and the single cornutus. But in *Teliphasa hamata* sp. n., the saccus is separated and the phallus has two cornuti. In addition, *Teliphasa amica* and *Teliphasa nubilosa* are also much alike in the male genitalia, and the differences between them are stated under *Teliphasa nubilosa*.

#### Description.

Adult (Fig. 12): Wingspan 36.0–40.0 mm.

#### Distribution.

China (Fujian, Guangxi, Hebei, Henan, Hubei, Jiangxi, Sichuan, Shandong, Tianjin, Yunnan, Zhejiang, Taiwan), Korea, Japan.

### Teliphasa sakishimensis

Taxon classificationAnimaliaLepidopteraPyralidae

Inoue & Yamanaka, 1975

Teliphasa sakishimensis Inoue & Yamanaka, 1975: 100. Type Locality: Japan (Mt. Banna).

#### Distribution.

China (Hubei, Sichuan, Taiwan), Japan.

#### Remarks.

Inoue and Yamanaka (1975) described this species from Taiwan in detail. Song and He (1977) reported this species occuring in Hubei and Sichuan provinces from Chinese Mainland. We are unable to check this species in our study, but *Teliphasa sakishimensis* can be easily separated by having a clear thorn-like projection from the juxta (Inoue and Yamanaka, 1975).

## Funds

This study was supported by the National Natural Science Foundation of China (No. 31272356) and the Research Fund for the Doctoral Program of Higher Education (No. 20130031110008).

## Supplementary Material

XML Treatment for Teliphasa

XML Treatment for Teliphasa spinosa

XML Treatment for Teliphasa similalbifusa

XML Treatment for Teliphasa erythrina

XML Treatment for Teliphasa hamata

XML Treatment for Teliphasa nubilosa

XML Treatment for Teliphasa albifusa

XML Treatment for Teliphasa elegans

XML Treatment for Teliphasa amica

XML Treatment for Teliphasa sakishimensis
